# Metabolic Plasticity and Virulence-Associated Factors of *Sporothrix brasiliensis* Strains Related to Familiar Outbreaks of Cat-to-Human Transmitted Sporotrichosis

**DOI:** 10.3390/jof9070724

**Published:** 2023-07-04

**Authors:** Dario Corrêa-Junior, Iara Bastos de Andrade, Vinicius Alves, Igor Avellar-Moura, Tânia Rodrigues-Alves, Vanessa Brito de Souza Rabello, Glauber R. de S. Araújo, Luana Pereira Borba-Santos, Rosely Maria Zancopé-Oliveira, Rodrigo Almeida-Paes, Susana Frases

**Affiliations:** 1 Laboratório de Biofísica de Fungos, Instituto de Biofísica Carlos Chagas Filho, Universidade Federal do Rio de Janeiro, Rio de Janeiro 21941-902, Braziltaniarodrigues@biof.ufrj.br (T.R.-A.);; 2 Laboratório de Biologia Celular de Fungos, Instituto de Biofísica Carlos Chagas Filho, Universidade Federal do Rio de Janeiro, Rio de Janeiro 24020-141, Brazil; 3 Laboratório de Micologia, Instituto Nacional de Infectologia Evandro Chagas, Fundação Oswaldo Cruz, Rio de Janeiro 21041-210, Brazil; 4 Rede Micologia RJ, FAPERJ, Rio de Janeiro 20020-000, Brazil

**Keywords:** sporotrichosis, enzymes, thermotolerance, virulence, one health

## Abstract

*Sporothrix brasiliensis* is the main agent of zoonotic sporotrichosis transmitted by domestic cats in South America. In humans, sporotrichosis commonly presents with cutaneous or lymphocutaneous lesions, and in cats, with multiple ulcerated skin lesions associated with enlarged lymph nodes and respiratory signs. Fungal virulence factors may affect the clinical presentation of the mycoses. *Sporothrix* spp. present some virulence factors. This study aims to compare 24 *S. brasiliensis* strains from 12 familiar outbreaks of cat-to-human transmitted sporotrichosis. Fungal growth in different substrates, thermotolerance, resistance to oxidative stress, and production of enzymes were evaluated. An invertebrate model of experimental infection was used to compare the virulence of the strains. The strains grew well on glucose and *N*-acetyl-*D*-glucosamine but poorly on lactate. Their thermotolerance was moderate to high. All strains were susceptible to hydrogen peroxide, and the majority produced hemolysins but not phospholipase and esterase. There was no significant difference in the putative virulence-associated factors studied among the different hosts. Moreover, strains isolated from a human and a cat from four familiar outbreaks presented a very similar profile of expression of these factors, reinforcing the zoonotic transmission of *S. brasiliensis* in Brazil and demonstrating the plasticity of this species in the production of virulence factors.

## 1. Introduction

Species of the genus *Sporothrix* include dimorphic fungi, which inhabit the soil and decaying plants and can also be pathogenic to animals [1]. They belong to the Kingdom Fungi, Division Ascomycota, Class Pyrenomycetes, Order Ophiostomatales, and Family Ophiostomataceae, with about 53 species, including several species of medical and environmental relevance, such as *Sporothrix schenckii*, *Sporothrix luriei Sporothrix albicans*, *S. brasiliensis*, *Sporothrix globosa*, *Sporothrix mexicana*, *Sporothrix pallida*, *Sporothrix Brunneoviolacea*, and *Sporothrix dimorphospora* [2]. It is known that contact with decaying plant material is frequently observed in sporotrichosis cases due to *S. globosa* or *S. schenckii*, while *S. brasiliensis*-associated sporotrichosis is more related to zoonotic transmission by naturally infected cats [1].

The saprophytic filamentous form, also observed in in vitro cultures incubated between 25 °C and 30 °C, presents hyaline septate hyphae, 1–2 μm wide, with conidiogenous cells that arise from differentiated hyphae and form conidia in groups, in small denticles, in a so-called sympodial arrangement. These unicellular conidia are teardrop-shaped to clavate [3] and do not form chains [4]. In their parasitic form, yeast cells are oval, unicellular, globular and/or cigar-shaped, between 2 and 8 µm in size, and may have one or more buds. They can be observed in direct tissue and/or histological examinations or from cultivation at 35 °C to 37 °C in an enriched culture medium, such as brain heart infusion agar (BHI) [5].

The largest known sporotrichosis epidemic associated with a common environmental source occurred in South Africa in the 1950s, involving 3000 mineworkers. In the 1980s, in the United States, especially in the Mississippi Valley, there was an outbreak of reforestation workers infected by pine trees and moss seedlings. In the early 1990s, a micro epidemic occurred with people infected by hay stored in an abandoned house [6]. The zoonotic transmission of sporotrichosis has been sporadically described as involving accidents with snakes and birds, as well as bites from mosquitoes, rats, horses, squirrels, and fish [7]. The importance of the cat in zoonotic transmission was first noted in a report involving five people exposed to a sick animal [8]. In Brazil, there are currently two important disease transmission routes for humans: a sapronotic route that involves direct contact with the soil and the decomposition of organic matter; and a zoonotic route, in which domestic cats actively participate in the transmission [9]. In the state of Rio de Janeiro, the emergence of zoonotic sporotrichosis was noticed in the late 1990s, and continues to this day, with hyperendemic levels [10]. Cases have been described throughout Brazil [11] and more recently in other South American countries [12,13] and imported cases in Europe [14].

In humans, sporotrichosis mostly presents as cutaneous or lymphocutaneous lesions, including papules, nodules, and ulcers. These lesions usually appear at the site of trauma a few days to several months after exposure [15]. In addition to the cutaneous presentations, *S. brasiliensis* can be associated with ocular involvement, disseminated disease, central nervous system disease, and hypersensitivity reactions [16]. In cats, the most common clinical manifestations include multiple ulcerated skin lesions associated with enlarged lymph nodes and the presence of respiratory signs [17]. Although human cases of disseminated sporotrichosis occur more commonly among people with immunosuppressive conditions [18], a similar association does not occur in feline sporotrichosis, since the sporotrichosis incidence does not increase in cats with feline immunodeficiency virus or feline leukemia virus [19].

Virulence is the relative capacity of a microbe to cause damage in a host. This definition broadly includes the enormous diversity of microbe–host interactions. Host damage can drive microbial processes, host immune response, or both, and disease occurs when host damage interferes with homeostasis [20]. Virulence factors enable and amplify the capacity for fungal growth into the host [21]. The main fungal virulence factors include metabolic and structural plasticity, which can cause clinical alterations in the mycoses caused by them. Thermotolerance, production of hydrolytic enzymes, and factors related to adhesion and initiation of infection are the main virulence factors observed in these pathogens. Enzymatic activities in fungi are essential for their nutrition, maintenance of their metabolic pathways, and are directly related to virulence [22]. Hydrolytic enzymes play a central role in fungal metabolism, causing damage to host cells and providing nutrients in a restricted and harsh environment. In addition, they act in the adhesion and survival of the pathogen on mucosal surfaces and during the invasion of host tissues [23].

It is presumed that the origin of the *Sporothrix* spp. virulence arose through inter-microbial interactions in their natural habitat [24]. *Sporothrix* spp. present some virulence factors described in the literature, such as glycoproteins, secreted proteins, extracellular vesicles, thermotolerance, ability to produce melanin, ergosterol peroxide, dimorphism [25], and more recently, the ability to form biofilm both in the filamentous and yeast forms [26]. Pathogenicity is closely related to virulence factors since they allow the establishment and development of the fungus in the host tissue. Therefore, it is necessary to study the metabolic plasticity of these agents to clarify questions related to their biology and participation in the infectious process. Here, we explored some virulence-associated factors of 24 *S. brasiliensis* strains from 12 familiar outbreaks of cat-to-human transmitted sporotrichosis that occurred in Rio de Janeiro, Brazil. This study assessed the relationships between virulence factors of strains isolated from humans and cats, the similarity of these factors on strains from a single outbreak, and whether all *S. brasiliensis* strains equally produce high levels of virulence-associated factors.

## 2. Materials and Methods

### 2.1. Paired Samples of Cats and Humans

Twenty-four *S. brasiliensis* strains from human patients and their respective cats with sporotrichosis treated at the Evandro Chagas National Institute of Infectious Diseases (INI-Fiocruz), previously identified by a species specific-PCR [27], were used (Table 1). Strains were obtained from the Coleção de Fungos Patogênicos, Fiocruz, Rio de Janeiro, Brazil (WDCM 951). All humans reported a history of contact with the sick cat before the onset of symptoms, as previously reported [28].

### 2.2. Inoculum Preparation

All *S. brasiliensis* strains were previously cultivated at 25 °C on potato dextrose agar (PDA—Difco, Franklin Lakes, NJ, USA) to obtain conidia, and the filamentous colonies were washed with phosphate-buffered saline (PBS) pH 7.2 ± 0.1, containing 15% glycerol. To obtain yeast cells, strains were cultivated on BHI agar (Difco, Franklin Lakes, NJ, USA) at 35 °C. Both incubations occurred for 7 days and yeasts were used in all experiments.

### 2.3. Evaluation of Fungal Growth

#### 2.3.1. Growth Assay on Different Carbon Sources

The growth of strains was evaluated in the presence of glucose, lactate, and *N*-acetyl-glucosamine (GlcNAc) as carbon sources, with an initial inoculum of 1 × 10^6^ yeasts/mL prepared in yeast nitrogen base medium (Difco, Franklin Lakes, NJ, USA) supplemented with 0.5% *w*/*v* lactate, 0.5% *w*/*v* GlcNAc, 0.5%, 1%, or 2% glucose (all from Sigma-Aldrich, Burlington, MA, USA). Aliquots of 100 µL were inoculated in triplicate in 96-well flat bottom plates (Jet Biofil, Guangzhou, China) and incubated at 35 °C for 7 days. The optical density of the wells at 530 nm was measured in a spectrophotometer (SpectraMax Plus 384, Molecular Devices, San Jose, CA, USA) every 24 h during the incubation period for the construction of growth curves [18].

#### 2.3.2. Yeast Growth Assay in Powdered Claw Suspension

Healthy cat claws were crushed with a mortar and pestle, sterilized by autoclavation, and subsequently mixed with a minimum medium (MM) containing 15 mM glucose, 10 mM MgSO_4_7·H_2_O, 29 mM KH_2_PO_4_, 13 mM glycine, and 3 µM thiamine (all compounds from Merck Millipore, Darmstadt, Germany) to form a suspension of 2% *w*/*v* powdered claws. An inoculum of 1 × 10^6^ yeast cells/mL was prepared in MM with or without (control) powdered claws and added to the wells of a 96-well plate in a 200 μL/well volume. Plates were incubated at 35 °C for 7 days, and the growth was analyzed by the absorbance measurement at 530 nm using a microplate reader (SpectraMax M3; Molecular Devices, San Jose, CA, USA). Mitochondrial activity was determined with the CyQUANT XTT assay (Thermo Fisher, Waltham, MA, USA). A mixture of XTT reagent and electron coupling reagent was prepared at a ratio of 6 to 1. Seventy microliters of the mixture were added to each well. Then, the plate was incubated at 37 °C for 4 h in the dark in a 5% CO_2_ incubator. The optical densities (ODs) were determined, in two technical replicates, using the SpectraMax^®^ Plus 384 Microplate Reader (SpectraMax M3; Molecular Devices, San Jose, CA, USA) at 450 nm and 660 nm. Mitochondrial activity was expressed by the decrease in OD (450–660 nm), discounting the average of the ODs of the controls [29].

### 2.4. Thermotolerance Evaluation

An inoculum of 1 × 10^6^ yeasts/mL was prepared from all strains. Then, 5 µL aliquots of the cell suspension were inoculated in triplicate in two Petri dishes (90 mm) containing PDA medium and incubated at 25 °C and 35 °C for a total period of 7 days. After the incubation, the diameter of the colonies was measured in millimeters, and then the percentage of growth inhibition (%GI) was calculated, as described [30]. The thermotolerance of each strain was classified as %GI from 0 to 33%—high thermotolerance, %GI from 34 to 65%—moderate thermotolerance, and %GI from 66 to 100%—low thermotolerance.

### 2.5. Resistance to Oxidative Stress

For the analysis of oxidative stress in *S. brasiliensis*, 57.5 mL of BHI medium (Difco, Franklin Lakes, NJ, USA), sterilized in an autoclave and cooled to 50 °C, was supplemented with 2.5 mL of a cell suspension (1 × 10^6^ yeasts/mL) in 0.45% NaCl solution. This suspension was added to a Petri dish (15 × 150 mm) and then gently shaken to obtain a homogeneous medium. After solidification, four wells of 5 mm in diameter and distributed equidistantly were made on each plate. In each well, 65 µL of 5% *v*/*v* hydrogen peroxide (Proquimios, Rio de Janeiro, Brazil) was applied. The assessment of resistance to oxidative stress was carried out by measuring the diameter of the fungal growth inhibition halo formed around each well, with the aid of a pachymeter, after 7 days of incubation at 35 °C [18].

### 2.6. Production of Extracellular Enzymes

#### 2.6.1. Urease Production

In a final volume of 2 mL of Christensen urea broth (peptone 0.1%, NaCl 0.5%, KH_2_PO_4_ 0.2%, urea 2%, glucose 0.1%, phenol red 0.0016%), 200 µL of a cell suspension containing 1 × 10^6^ yeasts/mL was added. These suspensions were incubated at 35 °C for 7 days, then centrifuged at 10,000× *g* for 10 min, and 100 µL of the supernatant was transferred in triplicate to a sterile 96-well flat-bottom polystyrene plate (Jet Biofil, Guangzhou, China). *Candida krusei* (ATCC 6258) and *Candida parapsilosis* (ATCC 22019) were used as negative controls and *Cryptococcus neoformans* (H99) as a positive control of urease production. The absorbance of the samples was measured in an Epoch Biotek spectrophotometer at 559 nm [31].

#### 2.6.2. Hemolytic, Phospholipase, and Esterase Production

Enzymatic activity was measured in Petri dishes (90 mm) containing specific media for testing each enzyme. Each enzymatic activity was determined through the Pz value Pz=AB, where A is the colony diameter in millimeters (mm) and B is the colony diameter (mm) plus the halo of enzymatic activity around the fungal colony. To prepare the inoculum, a cell suspension of 1 × 10^6^ yeasts/mL was used, where 5 µL aliquots were inoculated in triplicate on the surface of the undermentioned media and incubated for 7 days at 35 °C. Positive samples showed a halo around the colonies. *Candida albicans* (ATCC 18804) was used as a positive control to assess the quality of the culture media [22]. The enzymatic activity was classified into four categories as described: Pz: 1—Negative; Pz between 0.70 and 0.99—low activity; Pz between 0.40 and 0.69—moderate activity; and Pz lower than 0.39—high activity [32]. The hemolytic activity was determined by 5% Sheep Blood Agar (PlastLabor, Rio de Janeiro, Brazil) [22]. Egg Yolk Agar, composed of 2% glucose (Sigma–Aldrich, St Louis, MO, USA), 1% peptone, 0.5% yeast extract (Difco, Franklin Lakes, NJ, USA), 4% NaCl, 0.074% CaCl_2_ (Sigma–Aldrich, Burlington, MA, USA), 1.5% Agar (Difco, Franklin Lakes, NJ, USA), plus 8% organic egg yolk emulsion at pH 7.0, was used to determine phospholipase activity [32]. Tween Agar (1% peptone; 0.5% yeast extract; 0.01% CaCl_2_; 1.5% agar), plus 0.1% sterile Tween 80 (Sigma–Aldrich, Burlington, MA, USA), pH 7.0 was used to determine esterase activity [33].

### 2.7. Survival Tests on Molitor

For survival studies, we use the adapted methodology proposed by Lozoya-Pérez and collaborators [34], where larvae of at least 1 cm of uniform color, without dark spots or grayish marks, were selected. The animals were infected in the last left proleg with 10 μL of *S. brasiliensis* yeast cell suspensions (1 × 10^6^ yeasts/mL), using a Hamilton syringe (701 N, 26 caliber, 10 μL capacity), into the hemocoel. Then, animals were kept in Petri dishes of 14 cm at 37 °C. Mortality changes were recorded for 10 days. A total of 40 animals were included in each experimental group: 10 infected, 10 control, 10 animals untouched, and 10 inoculated only with PBS. The animal groups were under observation for up to 10 days, and live animals at the end of this period, along with those killed by the fungal inoculum, were decapitated with a sterile scalpel. The hemolymph was used to calculate the colony-forming units (CFUs) by serial dilutions that were incubated on Mycosel plates (Difco, Franklin Lakes, NJ, USA), at 28 °C for 72 h. The experiment was performed in triplicate.

### 2.8. Statistical Analyses

All experiments were performed in triplicate, in three independent experimental sets, except when strictly described before, and the results were expressed as mean ± standard deviation. The results were analyzed using the GraphPad Prism 9 software (La Jolla, San Diego, CA, USA), with *p*-values less than 0.05 to determine the significance. Non-parametric tests were used as appropriate and necessary in the different analyses and comparisons. The overall analysis of all virulence-related phenotypes was performed through a heat map constructed with the Heatmapper tool [35]. The correlation between the expression of different phenotypes by the same strain was assessed using Spearman’s correlation in GraphPad Prism 9 software.

## 3. Results

### 3.1. Fungal Growth on Different Substrates

**Figure 1:**
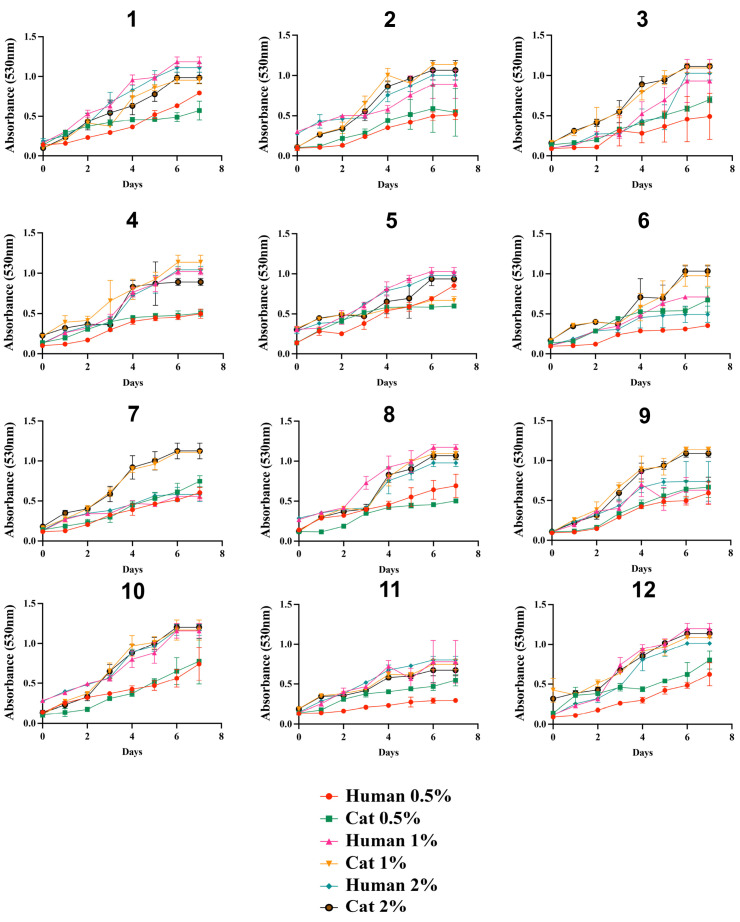
Growth curves of *Sporothrix brasiliensis* strains from 12 familiar outbreaks of sporotrichosis under 0.5%, 1%, and 2% glucose. The optical density (530 nm) of cultured strains was measured daily for 7 days. Results are expressed as the mean and standard deviation of readings obtained from three independent experiments performed in technical triplicates.

**Figure 2:**
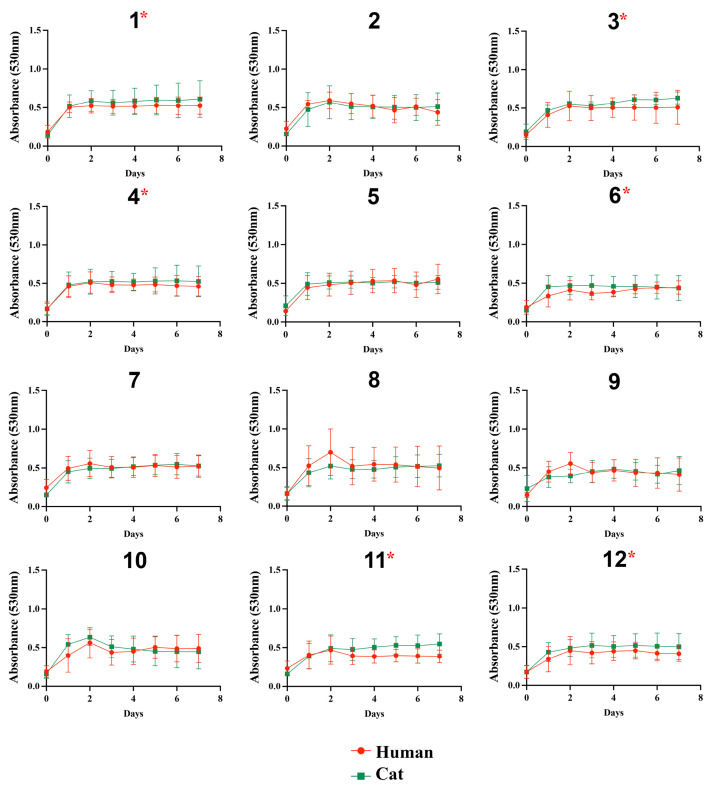
Growth curves of *Sporothrix brasiliensis* strains from 12 familiar outbreaks of sporotrichosis under 0.5% lactate. The optical density (530 nm) of cultured strains was measured daily for 7 days. Results are expressed as the mean and standard deviation of readings obtained from three independent experiments performed in technical triplicates. The asterisk symbol indicates a statistically significant difference between the growth curves of paired cases, *p*-value (Mann–Whitney test) ≤ 0.05.

**Figure 3:**
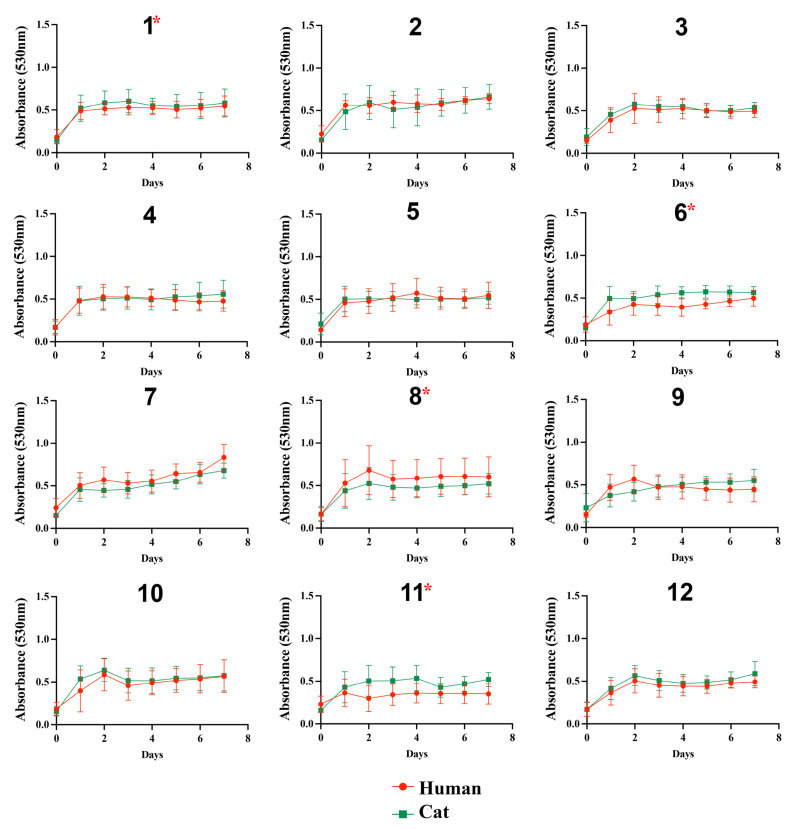
Growth curves of *Sporothrix brasiliensis* strains from 12 familiar outbreaks of sporotrichosis under 0.5% GlcNAc. The optical density (530 nm) of cultured isolates was measured daily for 7 days. Results are expressed as the mean and standard deviation of readings obtained from three independent experiments performed in technical triplicates. Asterisks indicate significant differences between growth curves of human- and cat-derived strains from the familiar outbreak. The asterisk symbol indicates a statistically significant difference between the growth curves of paired cases, *p*-value (Mann–Whitney test) ≤ 0.05.

The growth profile of the isolates in the presence of glucose was similar among the 24 *S. brasiliensis*, regardless of glucose concentrations (Figure 1). In general, all isolates grew poorly in the presence of lactate (Figure 2). The growth of the *S. brasiliensis* strains herein studied was more abundant in the presence of GlcNAc than lactate (Figure 3). Growth curves for human- and cat-derived *S. brasiliensis* strains from the familiar outbreaks were similar for all carbon sources evaluated; cases 1, 3, 4, 6, 11, and 12 presented significant growth differences on lactate and cases 1, 6, 8, and 11 on GlcNAc (*p*-value ≤ 0.05, Mann–Whitney test).

**Figure 4:**
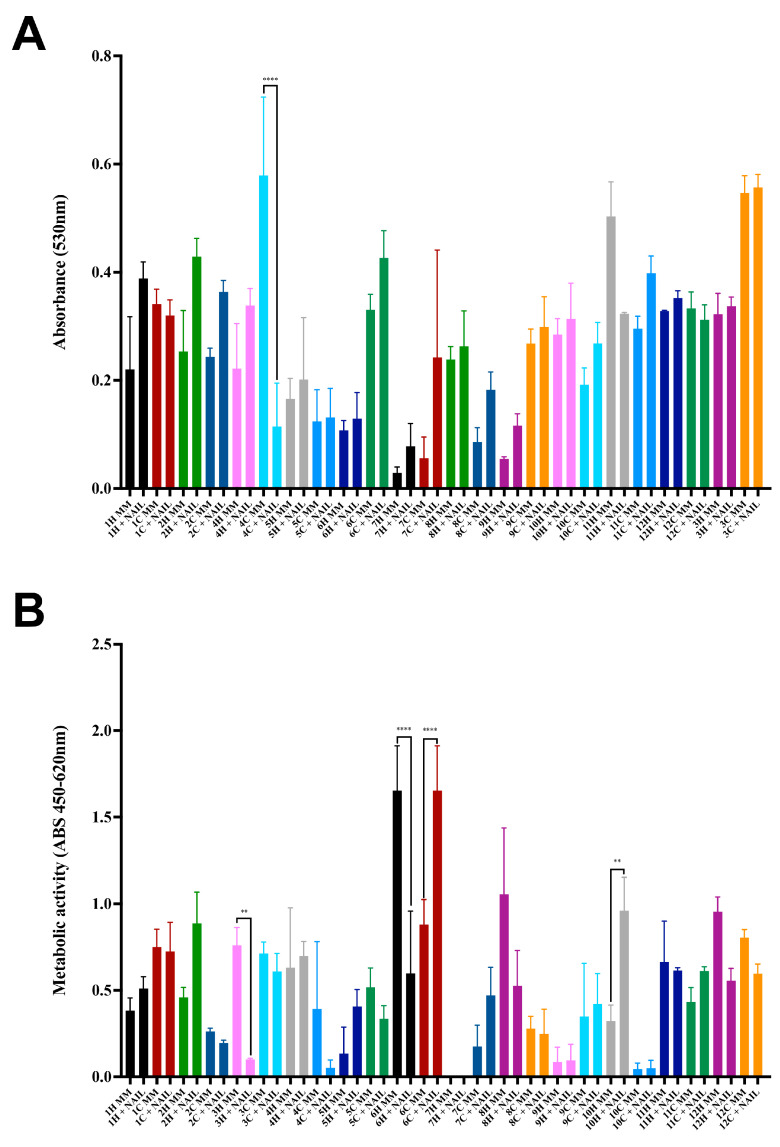
Growth of *Sporothrix brasiliensis* strains from 12 familiar outbreaks of sporotrichosis using cat claws as a nutrient. (**A**) Growth of *S. brasiliensis* in minimal medium (MM) and MM supplemented with 2% cat claws powder. (**B**) Metabolic activity of *S. brasiliensis* strains grown in MM and MM supplemented with 2% claws powder. Results are expressed as the mean plus standard deviation of three independent experiments performed in technical triplicates. Bar colors represent the different familiar outbreaks. The asterisk symbol indicates a statistically significant difference between the growth curves of paired cases, *p*-value (Tukey’s multiple comparisons test) ≤ 0.05.

The majority of *S. brasiliensis* strains exhibited better growth in the MM supplemented with powdered claws than in MM without any supplementation after 7 days of incubation (Figure 4A). Besides growth, metabolic activity after this incubation time was quantified by XTT-reaction assay (Figure 4B). The human *S. brasiliensis* strain from outbreak 7 did not have metabolic activity after this incubation period. The metabolic activity of human- and cat-derived strains was similar on both MM and MM supplemented with cat claw powder. In addition, outbreaks 3, 4, and 6 presented differences between growth in MM of human and cat strains (*p*-values = 0.0059, =0.0042, and <0.0001, respectively, Tukey’s multiple comparisons test).

### 3.2. Susceptibility to Physical and Chemical Stressors

**Figure 5:**
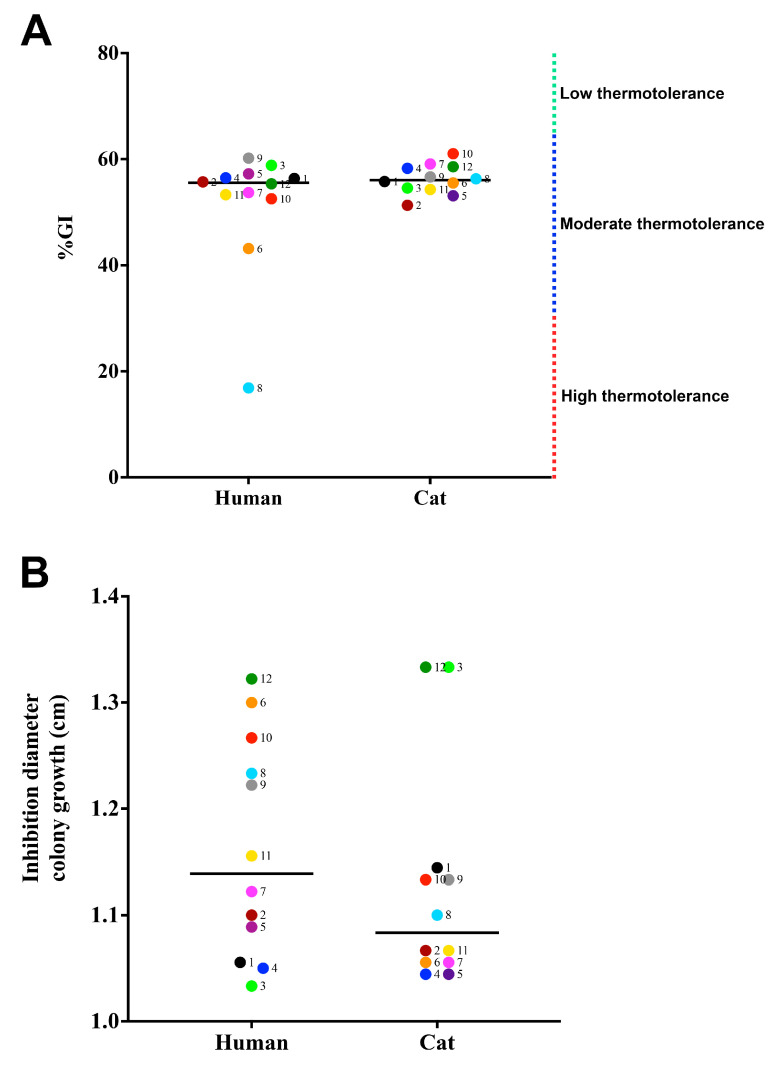
Resistance of *Sporothrix brasiliensis* strains from 12 familiar outbreaks of sporotrichosis against physical and chemical stressors. (**A**) Thermotolerance of *S. brasiliensis*, the smaller the percentage of growth inhibition (%GI), the greater the thermotolerance of the isolates. (**B**) Diameters in millimeters of growth inhibition of *S. brasiliensis* against exposure of yeast cells to 5% hydrogen peroxide. Each dot represents the mean value of three independent experiments performed in technical triplicates. Dot colors and numbers after dots represent the different familiar outbreaks. Black lines represent the median value of each group of strains.

All strains analyzed showed thermotolerance, ranging from high (n = 1) (%GI 0 to 33%) to moderate thermotolerance (n = 23) (%GI 34 to 65%). Cases 6 and 8 showed significant differences between human and animal isolates (Figure 5A) (*p*-values = 0.0313 and <0.0001, respectively, Mann–Whitney test). Figure 5B presents data about the oxidative stress of the clinical *S. brasiliensis* isolates herein studied. The oxygen-derived oxidant susceptibility profile of strains isolated from cats and humans varied (*p*-value ≤ 0.0001, Mann–Whitney test). Moreover, the paired strains from outbreak 6 showed a significant difference between them (*p*-value = 0.0370, Mann–Whitney test).

### 3.3. Production of Extracellular Enzymes

**Figure 6:**
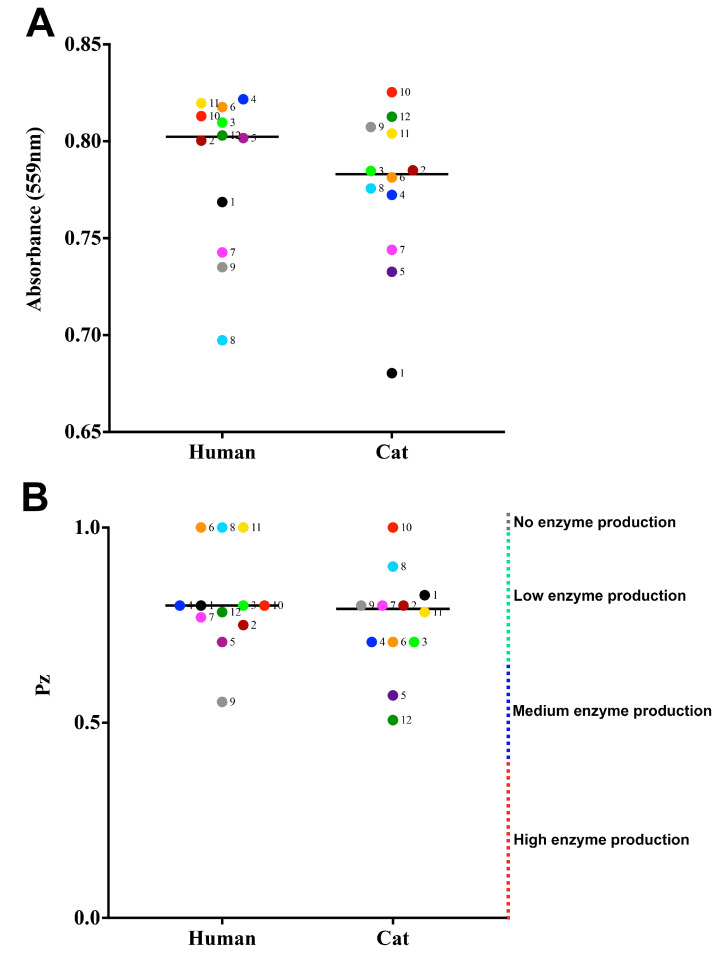
Production of extracellular enzymes by *Sporothrix brasiliensis* strains from 12 familiar outbreaks of sporotrichosis. Results are expressed as the mean and standard deviation of (**A**) urease and (**B**) hemolytic activity. Urease activity is expressed as the optical density (559 nm) of yeast *S. brasiliensis* cultures in Christensen’s urea broth after 7 days of growth at 35 °C. Hemolytic activity is expressed as the Pz of the enzyme of yeast *S. brasiliensis* cultures on sheep blood agar. Each dot represents the mean value of three independent experiments performed in technical triplicates. Dot colors and numbers after dots represent the different familiar outbreaks. Black lines represent the median value of each group of strains.

All samples produced urease, as visually determined by the change of the medium color to pink, like that observed for the *C. neoformans* strain and contrasting with that observed for the *C. parapsilosis* and *C. krusei* control strains that did not change the color of the Christensen’s urea broth. Outbreaks 1, 4, 5, 8, and 9 showed significant differences between human- and cat-derived strains (Figure 6A) (cases 1, 8, and 9 *p*-value ≤ 0.0001, 4 = 0.0238, and 5 = 0.0001, Tukey’s multiple comparisons test).

For the enzymes evaluated on agar plates, the control *C. albicans* strain yielded Pz values of 0.4, 0.6, and 0.6 for phospholipase, esterase, and hemolysin, respectively. There was no enzymatic production of phospholipase and esterase by the 24 *S. brasiliensis* strains of this study. Among these strains, four (16.7%), three isolated from humans and one isolated from a cat, from four distinct outbreaks (6H, 8H, 10C, and 11H) were unable to produce hemolysin (Figure 6B). The production of hemolysin varied from low enzymatic activity (n= 17) (Pz between 0.70 and 0.99) to moderate (n = 3) (Pz between 0.40 and 0.699). Differences between human- and cat-derived strains were not significant (*p*-value < 0.05, Tukey’s multiple comparisons test).

### 3.4. Survival Tests on Tenebrio Molitor

**Figure 7:**
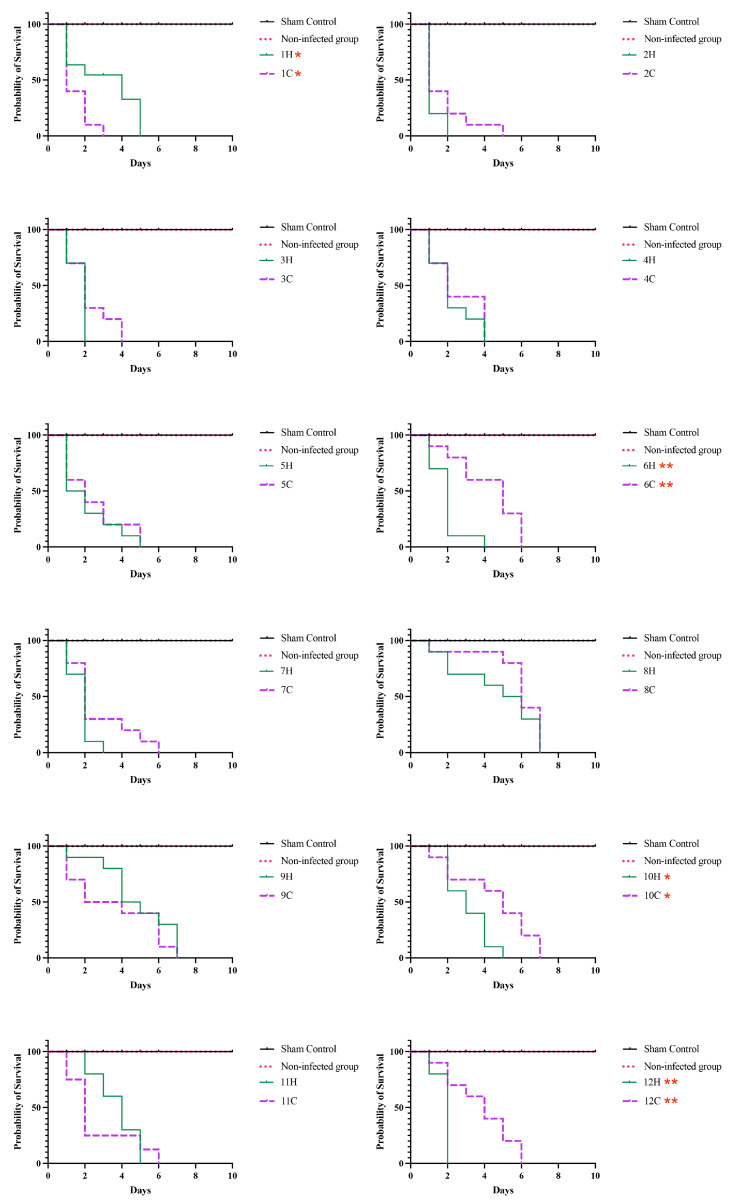
Survival curves of *Tenebrio molitor* larvae infected with *Sporothrix brasiliensis* strains from 12 familiar outbreaks of sporotrichosis. Larvae were infected with a 10 µL suspension containing 1 × 10^6^ yeast-like cells and incubated at 37 °C and observed until 10 days after inoculation. A total of 40 animals were included in each experimental group, and these were monitored daily to assess mortality, which was defined as a lack of irritability and the presence of extensive body melanization. The asterisk symbol indicates a statistically significant difference between the growth curves of paired cases, *p*-value (Log-rank (Mantel-Cox) test) ≤ 0.05.

Figure 7 illustrates the survival of larvae infected with the studied *S. brasiliensis* strains and incubated at 37 °C. *T. molitor* larvae showed median survival values ranging from 2 to 7 days. The Mantel–Cox test revealed a difference in survival patterns of human and cat-derived strains from outbreaks 1 (*p*-value = 0.0129), 6 (*p*-value = 0.0019), 10 (*p*-value = 0.0307), and 12 (*p*-value = 0.0042) (Log-rank Mantel–Cox test).

**Figure 8:**
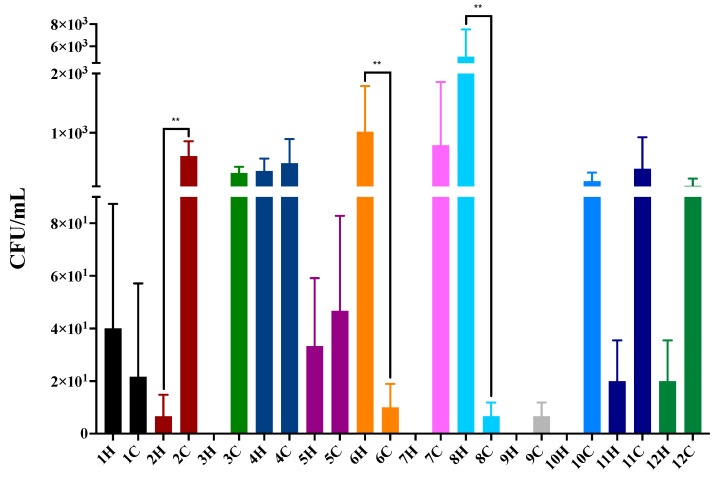
Colony-forming units (CFUs/mL) of *Sporothrix brasiliensis* in *Tenebrio molitor*. Results are expressed as the mean plus standard deviation of three independent experiments performed in technical triplicates. Bar colors represent the different familiar outbreaks. ** *p* = 0.0022, Mann–Whitney test.

To demonstrate fungal growth in inoculated animals, the colony-forming units (CFUs) were quantified in infected animals (Figure 8). Cases 2, 6, and 8 show differences in CFU compared to corresponding paired cases (*p* = 0.0022, Mann–Whitney test).

### 3.5. Comparative Analysis of Virulence-Related Factors

**Figure 9:**
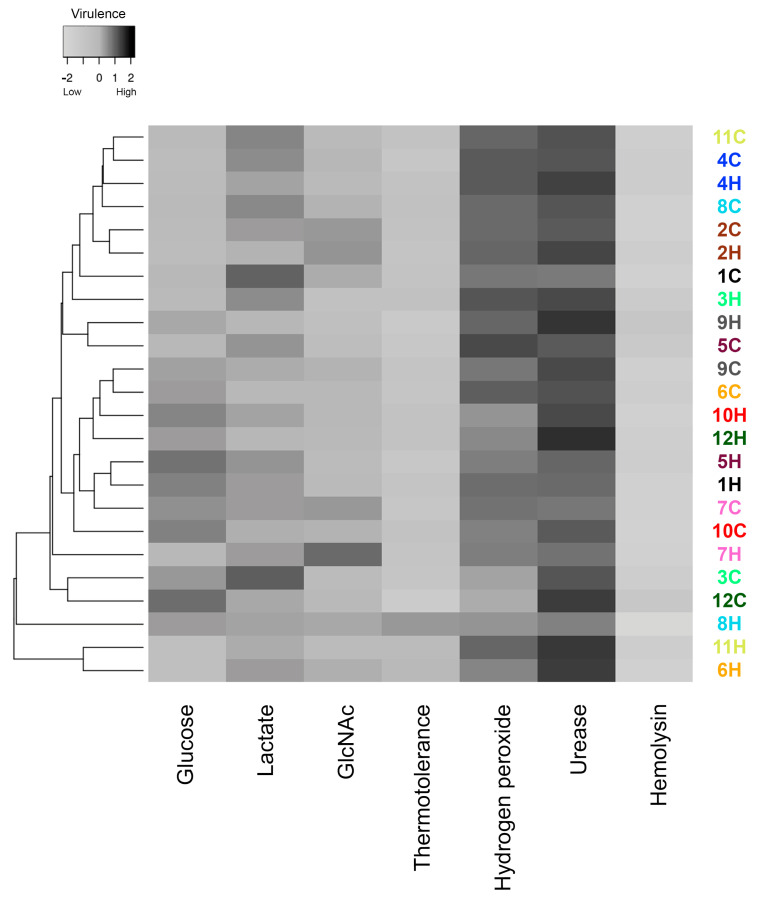
Global expression of putative virulence-associated factors by *Sporothrix brasiliensis* strains from 12 familiar outbreaks of sporotrichosis. The gray scale in the heatmap ranges from low (white) to high virulence (black). Different strains are represented in the lines of the heatmap, and the different virulence factors herein are studied in the columns of the heat map. Strains were grouped in a dendrogram reflecting the similarity between the virulence-related phenotypes of each strain. Colors of the strain identification numbers represent the different familiar outbreaks.

As depicted in Figure 9, none of the 24 *S. brasiliensis* strains presented a high level of all herein-studied putative virulence-associated factors. In general, the major phenotypic features potentially related to fungal virulence among the studied *S. brasiliensis* strains were urease production and susceptibility to hydrogen peroxide. The dendrogram clustered the strains according to their global similarity in the production of those studied factors. It is interesting to note that human- and cat-derived strains from outbreaks 2 and 4 clustered together, while strains from outbreaks 7 and 9 clustered very closely.

**Figure 10:**
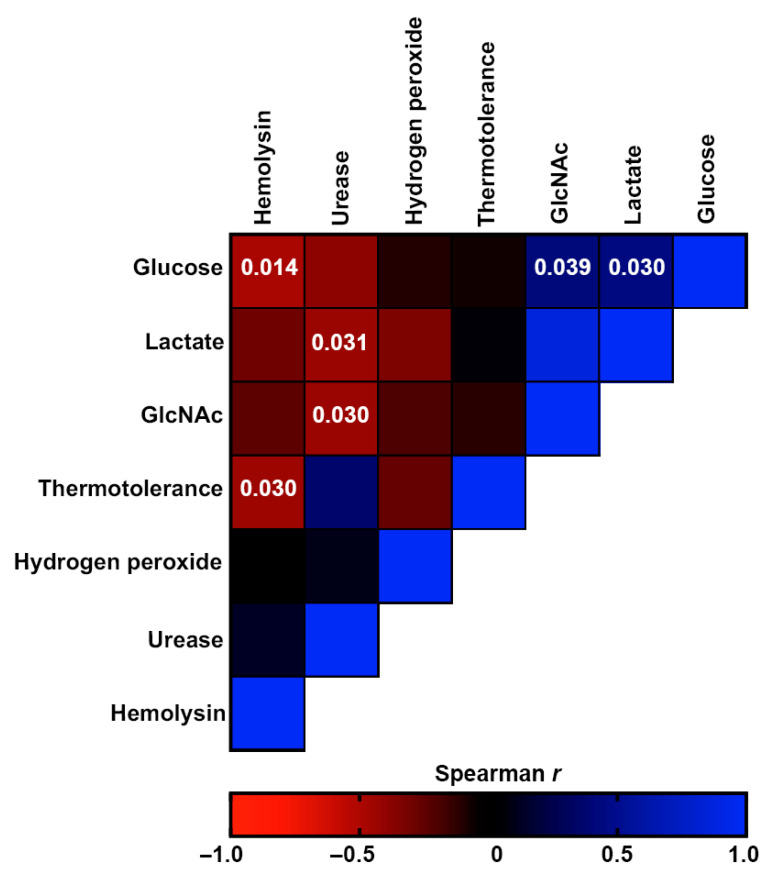
Correlation analyses of the virulence-associated factors produced by the *Sporothrix brasiliensis* strains from 12 familiar outbreaks of sporotrichosis. The heat map represents the Spearman’s correlation coefficients of the associations. The red color represents negative monotonic correlations, whereas the blue color represents positive monotonic correlations. Probability values of statistically significant correlations (*p* < 0.05) are displayed within the respective squares of the heat map.

Possible correlations between the different fungal phenotypes were also investigated. Figure 10 shows a heatmap representing the Spearman’s correlation coefficients of the associations between different phenotypes of the studied isolates. The red color represents negative associations, whereas the blue color represents positive associations. Growth on glucose was negatively monotonically related to growth on GlcNAc (*p*-value = 0.039) and lactate (*p*-value = 0.030). Hemolysin production was positively monotonically related to growth on glucose and thermotolerance (*p*-values = 0.014 and =0.030, respectively), while urease production was positively monotonically related to growth on lactate and GlcNAc (*p*-values = 0.031 and =0.030, respectively).

Finally, a regression analysis was conducted to estimate the relative contributions of the studied virulence factors to the survival of *T. molitor*. As shown in Table 2, none of the herein-studied virulence-associated factors presented a significant association with larvae survival.

## 4. Discussion

The *S. brasiliensis* strains herein studied showed differences in some phenotypical characteristics that have been described as related to virulence in other human pathogenic fungi [36,37,38,39,40]. These aspects should be studied from a “One Health” perspective since this species continuously passes through different mammalian hosts and environmental sources [41]. This was the reason that motivated us to compare these phenotypes on strains obtained from humans and the cats that have infected them. In general, no differences were observed on human- and cat-derived *S. brasiliensis* strains, and for some outbreaks, phenotypes were extremely close for the related strains, suggesting that one passage through a mammalian host does not significantly affect the studied phenotypes.

Adaptations to environmental stressors may compel some fungi to acquire abilities that can increase their virulence and survival, driving the emergence of new threats to human, animal, and environmental health [42]. To survive the adverse conditions during parasitism, pathogenic fungi must adapt to the harsh microenvironments of the host. They may display altered phenotypic characteristics due to the selection pressure, in a process known as microevolution, as demonstrated in fungi of medical interest, such as *Aspergillus fumigatus* [43], *C. neoformans* [44], and *C. albicans* [45]. The present study also shows that *S. brasiliensis* has metabolic plasticity and may also suffer microevolution depending on the host. This plasticity to adapt to various stress conditions imposed during protracted infections on different patients was also observed previously for patients with cryptococcosis [46] and sporotrichosis [18].

Pathogenic fungi could use various carbon sources for development and survival [47,48]. Disturbances in host homeostasis can cause differences in glucose concentrations in the body [48], impacting the pathogenesis of the mycoses. For instance, colonization by *Candida* spp. is associated with glycemic control in patients with diabetes because hyperglycemia increases salivary glucose levels, promoting pathogen growth in the oral cavity [48]. Studies indicate that *C. albicans* can colonize immunocompetent individuals, and its cells are able to quickly adapt to available carbon sources in the host. Lactate, glucose, and GlcNAc are some of the carbon sources available for pathogens in the human body [49,50]. The *S. brasiliensis* growth studied here was noticeable in glucose since this is the main carbon source used by fungi. Concerning alternative carbon sources, the growth was slightly greater in the presence of GlcNAc than lactate, like that observed previously by our group in a group of *S. brasiliensis* strains isolated from human patients with chronic disseminated disease [18]. The skin tropism of *S. brasiliensis* may explain its preferential use of GlcNAc over lactate. GlcNAc is a glucosamine derivative that is naturally produced by humans and, together with D-glucuronate, forms hyaluronic acid, which is present in several body tissues, such as joints and skin. On the other hand, lactate is a widely abundant compound in most host niches, and it is a carboxylic acid that, in the body, is mainly found in its ionic form. Lactate is a product of glycolysis and plays fundamental roles in skeletal muscle tissue [48].

The production of extracellular enzymes allows the hydrolysis of skin components and promotes the invasion of the pathogen into the host tissues [51]. Moreover, when fungi are phagocytosed, they induce the release of oxidative chemical mediators, such as hydrogen peroxide, that cause oxidative stress in the microbe. Fungi, as an escape mechanism, produce catalases promoting fungal multiplication within macrophages [52]. The enzyme converts H_2_O_2_ into water and oxygen, allowing the fungus to remain viable after the host’s immune response, triggering its multiplication process [53]. The *S. brasiliensis* strains studied here were inhibited by hydrogen peroxide. Our results do not allow quantifying the catalase amount produced by the strains, but they show that they produce high amounts of catalase, due to the halo of growth inhibition lower than that described for *A. fumigatus* [54] and *S. brasiliensis* strains from chronic disease [18]. *S. brasiliensis* is an extracellular pathogen; therefore, its survival does not strongly depend on resistance to phagocytosis.

Phospholipase and esterase activities were not detected on the *S. brasiliensis* strains under the current study conditions. It is interesting to note that we previously detected esterase production in patients with chronic sporotrichosis [18], indicating that this enzyme may have a role in fungal maintenance in hosts with compromised immunity over extended periods of time. All isolates were able to produce urease. This enzyme is responsible for the use of urea as a source of nitrogen, causing the release of ammonia, resulting in a partial increase in the acidic pH [55]. In *C. neoformans*, urease is important for the invasion of the central nervous system since it facilitates the crossing of the blood–brain barrier [56]. It is known that *S. brasiliensis* produces higher levels of urease when compared to *S. schenckii* [31], and that *S. brasiliensis* could cause meningitis in severely immunosuppressed patients [57], which may be linked to the production of this enzyme. Interestingly, the urease levels produced by our strains were similar to that produced by the control *C. neoformans* strain, a traditional agent of fungal meningitis. Fungi can also secrete hemolysins responsible for the lysis of nucleated and red blood cells [58]. Four strains of the present study were unable to produce hemolysin. The production of this enzyme by *S. brasiliensis* isolates from human disseminated disease is useful during hematogenic dissemination [18]. Sporotrichosis in cats is progressive and blood dissemination will likely occur without proper management [59]. These proteins are important for the survival of microorganisms and are related to iron acquisition; therefore, their production is essential for the establishment of an infectious process [60].

*Sporothrix* spp. can be isolated from claw surfaces of naturally infected cats [61] and in an ex vivo biofilm model using the filamentous form of *Sporothrix* species and cat claws [26]. Our results show that the yeast-like form of *S. brasiliensis* can also use nutrients from cat claws to improve fungal growth, like that described in the literature [29]. This is important information, supporting that this species can survive on cat claws in its parasitic form, facilitating the zoonotic transmission of sporotrichosis. It is not possible to conclude that the added keratin facilitated the formation of biofilms, as the nail powder used may contain various nutrients for fungi. Furthermore, the low metabolic activity observed in certain *Sporothrix* strains may be related to this. To explore this further, future experiments may focus on identifying components present in cat nails that may promote the growth of *S. brasiliensis*.

The mealworm, also known as *T. molitor*, is an invertebrate animal model used to study fungal virulence [62]. *T. molitor* has been used to assess the virulence of entomopathogenic fungi such as *Metarhizium robertsii* [63], *Metarhizium brunneum* [64], and *Beauveria bassiana* [65] and human pathogens such as *C. albicans* [62], *C. neoformans* [62], and *Fonsecaea* spp. [66]. This study demonstrated the virulence of *S. brasiliensis* in these animals, with the median survival ranging from 2 to 7 days at 37 °C. The results that we have obtained for the yeast form are like that previously described for this species [34]. The observed outcomes concerning the yeast form align with previous reports on this particular species’ behavior.

The present study suggests that *S. brasiliensis* has a complex phenotype expression profile to adapt to the conditions found in the hosts. The data obtained in this study demonstrated that the *S. brasiliensis* isolates could grow in different nutrient sources available in hosts, present thermotolerance, susceptibility to the stress generated by hydrogen peroxide, and produce hemolysin. These metabolic factors may be associated with the installation/infection of *S. brasiliensis* in humans and other animals, demonstrating the plasticity of *S. brasiliensis* strains. The clinical signs of human and cat patients infected by this fungus are most probably linked to the host immunity or to other enzymes and virulence factors not evaluated in this study because they did not demonstrate significance with the survival of the experimental model herein used.

## Figures and Tables

**Table 1:** Paired samples of *Sporothrix brasiliensis* obtained at Fiocruz (Rio de Janeiro, Brazil), between 1998 and 2001 [28].

Case	Original Number	Host	Strain Identification
1	15485-1	Human	1H
	34-1	Cat	1C
2	15647-1	Human	2H
	47	Cat	2C
3	19536	Human	3H
	856	Cat	3C
4	16393-2	Human	4H
	92-2	Cat	4C
5	16415	Human	5H
	99-2	Cat	5C
6	16459	Human	6H
	75-1	Cat	6C
7	16672	Human	7H
	142-1	Cat	7C
8	17878	Human	8H
	98-2	Cat	8C
9	16910-1	Human	9H
	195-1	Cat	9C
10	17500	Human	10H
	165-1	Cat	10C
11	19182	Human	11H
	721-1	Cat	11C
12	19481	Human	12H
	792-1	Cat	12C

**Table 2:** Hierarchical regression analysis results for initial 24-strain data set.

Virulence Factor(s)	Correlation with Time to Death (*r*)	*R*^2^ Change	df	*F* Value	*p* Value
Glucose	4765	0.3199	1	4765	0.0443
Lactate	−7434	0.2641	1	4202	0.0571
NAG	−3996	0.2695	1	1879	0.1894
Thermotolerance	1144	0.4754	1	0.05934	0.8106
Hydrogen peroxide	−0.5222	0.1866	1	0.01536	0.9029
Urease	−8150	0.4145	1	1220	0.2858
Hemolysin	−3454	0.3832	1	1480	0.2399

## Data Availability

The data in this study are available in the presented manuscript.
